# 1-[4-(3,5-Difluoro­benz­yloxy)-2-hy­droxy­phen­yl]ethanone

**DOI:** 10.1107/S1600536810033787

**Published:** 2010-08-28

**Authors:** Ya-Tuan Ma, An-Ling Zhang, Mao-Sen Yuan, Jin-Ming Gao

**Affiliations:** a College of Science, Northwest A&F University, Yangling 712100, People’s Republic of China

## Abstract

The title compound, C_15_H_12_F_2_O_3_, has been obtained by the reaction of 2,4-dihy­droxy­lacetonephenone, potassium carbonate and 3,5-difluoro­benzyl bromide. The hy­droxy group is involved in an intra­molecular O—H⋯O hydrogen bond in each of the two independent mol­ecules in the asymmetric unit. The dihedral angle between the aromatic rings is  0.5 (2)° in one molecule and 1.9 (2)° in the other. In the crystal, weak C—H⋯O inter­actions link the mol­ecules into tetra­meric units aligned perpendicular to *b*.

## Related literature

For background to the Williamson reaction in organic synthesis, see: Dermer (1934 ▶). For a related structure, see: Ma *et al.* (2010 ▶).
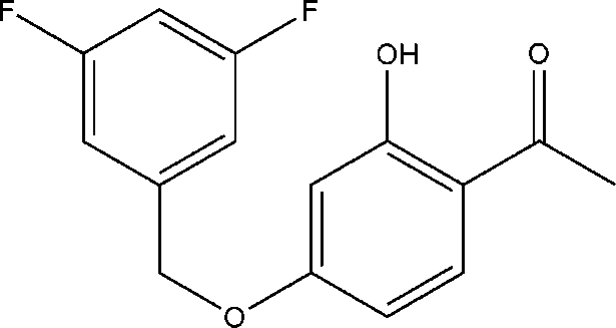


## Experimental

### 

#### Crystal data

C_15_H_12_F_2_O_3_
*M* *_r_* = 278.25
Triclinic, 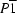

*a* = 7.4220 (8) Å
*b* = 13.0329 (14) Å
*c* = 14.1171 (16) Å
α = 83.921 (2)°
β = 77.913 (1)°
γ = 76.501 (1)°
*V* = 1296.1 (2) Å^3^
*Z* = 4
Mo *K*α radiation
μ = 0.12 mm^−1^
*T* = 298 K
0.40 × 0.32 × 0.28 mm

#### Data collection

Siemens SMART CCD area-detector diffractometer
Absorption correction: multi-scan (*SADABS*; Sheldrick, 1996 ▶) *T*_min_ = 0.955, *T*_max_ = 0.968
6817 measured reflections
4491 independent reflections
2244 reflections with *I* > 2σ(*I*)
*R*_int_ = 0.036

#### Refinement

*R*[*F* ^2^ > 2σ(*F* ^2^)] = 0.072
*wR*(*F* ^2^) = 0.231
*S* = 0.96
4491 reflections
363 parameters
H-atom parameters constrained
Δρ_max_ = 0.27 e Å^−3^
Δρ_min_ = −0.22 e Å^−3^

### 

Data collection: *SMART* (Siemens, 1996 ▶); cell refinement: *SAINT* (Siemens, 1996 ▶); data reduction: *SAINT*; program(s) used to solve structure: *SHELXS97* (Sheldrick, 2008 ▶); program(s) used to refine structure: *SHELXL97* (Sheldrick, 2008 ▶); molecular graphics: *SHELXTL* (Sheldrick, 2008 ▶); software used to prepare material for publication: *SHELXTL*.

## Supplementary Material

Crystal structure: contains datablocks I, global. DOI: 10.1107/S1600536810033787/bv2156sup1.cif

Structure factors: contains datablocks I. DOI: 10.1107/S1600536810033787/bv2156Isup2.hkl

Additional supplementary materials:  crystallographic information; 3D view; checkCIF report

## Figures and Tables

**Table 1:** Hydrogen-bond geometry (Å, °)

*D*—H⋯*A*	*D*—H	H⋯*A*	*D*⋯*A*	*D*—H⋯*A*
O2—H2⋯O1	0.82	1.81	2.533 (4)	147
O5—H5⋯O4	0.82	1.80	2.525 (4)	147
C8—H8⋯O5	0.93	2.49	3.382 (5)	161
C13—H13⋯O1^i^	0.93	2.44	3.342 (5)	165
C28—H28⋯O4^ii^	0.93	2.40	3.315 (5)	168

Symmetry codes: (i) 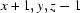; (ii) 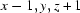.

**Fig. 1.**
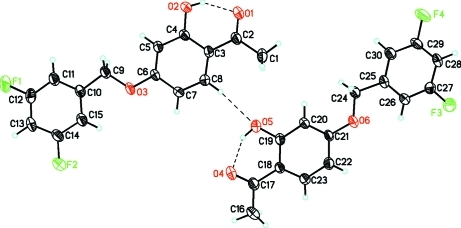
The molecular structure of (I). Displacement ellipsoids are drawn at the 30% probability level. Both the C—H···O interaction and the intramolecular hydrogen bonds are shown by dashed lines.

**Fig. 2.**
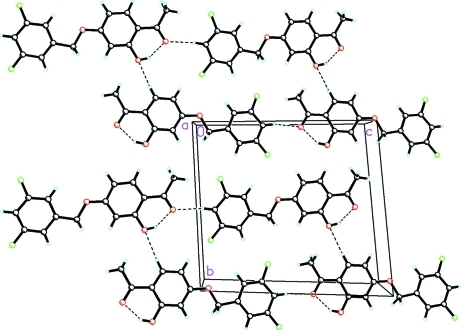
The packing viewed down the b axis showing the tetrameric units linked by C—H···O interactions. Both these interactions and the intramolecular hydrogen bonds are shown by dashed lines.

## supplementary crystallographic information

### Comment

The Williamson reaction is a very useful transformation in organic synthesis since the products are of value in both industrial and academic applications. It usually involves the employment of an alkali-metal salt of the hydroxy compound and an alkylhalide (Dermer, 1934).

In this paper, we present the title compound, (I), which was synthesized by the reaction of 2,4-dihydroxylacetonephenone, potassium carbonate and 3,5-difluorobenzyl bromide. In (I) (Fig. 1), the bond lengths and angles are normal and the dihedral angle between the aromatic rings is 0.51 (4)°. In addition to the intramolecular O—H···O hydrogen bonds, there are weak C—H···O interactions which link the molecules into tetrameric units aligned perpendicular to *b* (see Fig. 2).

### Experimental

2,4-Dihydroxylacetonephenone (4 mmol), potassium carbonate (8 mmol), 3,5-difluorobenzyl bromide (4 mmol), and 40 ml acetone were mixed in 100 ml flask. After 3 h stirring at 331 K, the crude product was obtained. The crystals were obtained by recrystallization from n-hexane/ethyl acetate.

### Refinement

The positions of all H atoms were fixed geometrically and distance to H atoms were set by the program, with C—H distance in the range 0.93–0.97 Å and O—H distance of 0.82 Å.

### Crystal data

**Table:**

C_15_H_12_F_2_O_3_	*Z* = 4
*M**_r_* = 278.25	*F*(000) = 576
Triclinic, *P*1	*D*_x_ = 1.426 Mg m^−^^3^
Hall symbol: -P 1	Mo *K*α radiation, λ = 0.71073 Å
*a* = 7.4220 (8) Å	Cell parameters from 1571 reflections
*b* = 13.0329 (14) Å	θ = 2.4–23.0°
*c* = 14.1171 (16) Å	µ = 0.12 mm^−^^1^
α = 83.921 (2)°	*T* = 298 K
β = 77.913 (1)°	Triclinic, colorless
γ = 76.501 (1)°	0.40 × 0.32 × 0.28 mm
*V* = 1296.1 (2) Å^3^	

### Data collection

**Table:**

Siemens SMART CCD area-detector diffractometer	4491 independent reflections
Radiation source: fine-focus sealed tube	2244 reflections with *I* > 2σ(*I*)
graphite	*R*_int_ = 0.036
ω scans	θ_max_ = 25.0°, θ_min_ = 1.5°
Absorption correction: multi-scan (*SADABS*; Sheldrick, 1996)	*h* = −8→8
*T*_min_ = 0.955, *T*_max_ = 0.968	*k* = −13→15
6817 measured reflections	*l* = −16→16

### Refinement

**Table:**

Refinement on *F*^2^	Primary atom site location: structure-invariant direct methods
Least-squares matrix: full	Secondary atom site location: difference Fourier map
*R*[*F*^2^ > 2σ(*F*^2^)] = 0.072	Hydrogen site location: inferred from neighbouring sites
*wR*(*F*^2^) = 0.231	H-atom parameters constrained
*S* = 0.96	*w* = 1/[σ^2^(*F*_o_^2^) + (0.1234*P*)^2^] where *P* = (*F*_o_^2^ + 2*F*_c_^2^)/3
4491 reflections	(Δ/σ)_max_ < 0.001
363 parameters	Δρ_max_ = 0.27 e Å^−^^3^
0 restraints	Δρ_min_ = −0.22 e Å^−^^3^

### Special details

**Table:**

Geometry. All e.s.d.'s (except the e.s.d. in the dihedral angle between two l.s. planes) are estimated using the full covariance matrix. The cell e.s.d.'s are taken into account individually in the estimation of e.s.d.'s in distances, angles and torsion angles; correlations between e.s.d.'s in cell parameters are only used when they are defined by crystal symmetry. An approximate (isotropic) treatment of cell e.s.d.'s is used for estimating e.s.d.'s involving l.s. planes.
Refinement. Refinement of *F*^2^ against ALL reflections. The weighted *R*-factor *wR* and goodness of fit *S* are based on *F*^2^, conventional *R*-factors *R* are based on *F*, with *F* set to zero for negative *F*^2^. The threshold expression of *F*^2^ > σ(*F*^2^) is used only for calculating *R*-factors(gt) *etc*. and is not relevant to the choice of reflections for refinement. *R*-factors based on *F*^2^ are statistically about twice as large as those based on *F*, and *R*- factors based on ALL data will be even larger.

### Fractional atomic coordinates and isotropic or equivalent isotropic displacement parameters (Å^2^)

**Table:**

	*x*	*y*	*z*	*U*_iso_*/*U*_eq_	
F1	1.1302 (5)	−0.2012 (2)	−0.40710 (17)	0.1012 (10)	
F2	1.0857 (4)	0.1549 (2)	−0.36867 (17)	0.0938 (10)	
O1	0.3966 (4)	−0.0294 (2)	0.39116 (18)	0.0685 (9)	
O2	0.5359 (4)	−0.1407 (2)	0.24593 (18)	0.0660 (9)	
H2	0.4839	−0.1278	0.3020	0.099*	
O3	0.7935 (4)	0.0311 (2)	−0.04745 (16)	0.0550 (8)	
C1	0.3850 (7)	0.1531 (3)	0.3919 (3)	0.0710 (13)	
H1A	0.3360	0.1414	0.4597	0.106*	
H1B	0.2913	0.2023	0.3626	0.106*	
H1C	0.4958	0.1815	0.3844	0.106*	
C2	0.4350 (6)	0.0503 (3)	0.3436 (3)	0.0537 (10)	
C3	0.5232 (5)	0.0463 (3)	0.2413 (2)	0.0429 (9)	
C4	0.5740 (5)	−0.0511 (3)	0.1963 (2)	0.0450 (9)	
C5	0.6623 (5)	−0.0580 (3)	0.1002 (2)	0.0457 (9)	
H5A	0.6932	−0.1227	0.0719	0.055*	
C6	0.7044 (5)	0.0296 (3)	0.0468 (2)	0.0425 (9)	
C7	0.6561 (5)	0.1271 (3)	0.0888 (2)	0.0499 (10)	
H7	0.6832	0.1871	0.0522	0.060*	
C8	0.5688 (5)	0.1336 (3)	0.1839 (2)	0.0486 (10)	
H8	0.5387	0.1987	0.2113	0.058*	
C9	0.8450 (6)	−0.0657 (3)	−0.0942 (2)	0.0511 (10)	
H9A	0.7336	−0.0936	−0.0915	0.061*	
H9B	0.9319	−0.1171	−0.0610	0.061*	
C10	0.9362 (5)	−0.0474 (3)	−0.1973 (2)	0.0453 (9)	
C11	0.9919 (6)	−0.1327 (3)	−0.2559 (3)	0.0585 (11)	
H11	0.9724	−0.1990	−0.2309	0.070*	
C12	1.0756 (6)	−0.1181 (4)	−0.3505 (3)	0.0630 (12)	
C13	1.1114 (6)	−0.0235 (4)	−0.3913 (3)	0.0592 (11)	
H13	1.1714	−0.0155	−0.4556	0.071*	
C14	1.0529 (6)	0.0592 (3)	−0.3312 (3)	0.0582 (11)	
C15	0.9681 (6)	0.0493 (3)	−0.2359 (3)	0.0543 (10)	
H15	0.9327	0.1073	−0.1978	0.065*	
F3	−0.1980 (4)	0.6542 (2)	0.85834 (18)	0.1013 (10)	
F4	−0.0009 (4)	0.2932 (2)	0.93069 (17)	0.0972 (10)	
O4	0.6255 (5)	0.4794 (2)	0.11718 (18)	0.0730 (9)	
O5	0.5718 (4)	0.3646 (2)	0.27167 (18)	0.0697 (9)	
H5	0.6143	0.3793	0.2147	0.104*	
O6	0.1814 (4)	0.5295 (2)	0.55010 (16)	0.0569 (8)	
C16	0.4955 (7)	0.6621 (4)	0.1001 (3)	0.0734 (14)	
H16A	0.5610	0.6523	0.0343	0.110*	
H16B	0.5433	0.7121	0.1277	0.110*	
H16C	0.3630	0.6883	0.1010	0.110*	
C17	0.5250 (6)	0.5590 (3)	0.1580 (3)	0.0551 (11)	
C18	0.4401 (5)	0.5514 (3)	0.2605 (2)	0.0426 (9)	
C19	0.4657 (6)	0.4531 (3)	0.3139 (2)	0.0487 (10)	
C20	0.3820 (5)	0.4430 (3)	0.4103 (2)	0.0476 (10)	
H20	0.3995	0.3774	0.4441	0.057*	
C21	0.2719 (5)	0.5314 (3)	0.4563 (2)	0.0425 (9)	
C22	0.2473 (6)	0.6292 (3)	0.4062 (2)	0.0519 (10)	
H22	0.1748	0.6886	0.4377	0.062*	
C23	0.3298 (5)	0.6385 (3)	0.3104 (2)	0.0501 (10)	
H23	0.3119	0.7047	0.2775	0.060*	
C24	0.2003 (6)	0.4312 (3)	0.6062 (2)	0.0500 (10)	
H24A	0.3325	0.4014	0.6070	0.060*	
H24B	0.1513	0.3818	0.5772	0.060*	
C25	0.0940 (5)	0.4486 (3)	0.7071 (2)	0.0452 (9)	
C26	−0.0051 (6)	0.5473 (3)	0.7369 (3)	0.0533 (10)	
H26	−0.0067	0.6063	0.6936	0.064*	
C27	−0.1003 (6)	0.5569 (3)	0.8303 (3)	0.0602 (12)	
C28	−0.1032 (6)	0.4739 (3)	0.8982 (3)	0.0573 (11)	
H28	−0.1689	0.4821	0.9616	0.069*	
C29	−0.0026 (6)	0.3781 (3)	0.8659 (3)	0.0575 (11)	
C30	0.0951 (6)	0.3632 (3)	0.7736 (3)	0.0564 (11)	
H30	0.1619	0.2962	0.7556	0.068*	

### Atomic displacement parameters (Å^2^)

**Table:**

	*U*^11^	*U*^22^	*U*^33^	*U*^12^	*U*^13^	*U*^23^
F1	0.142 (3)	0.099 (2)	0.0589 (15)	−0.0326 (19)	0.0144 (17)	−0.0386 (15)
F2	0.127 (3)	0.0745 (19)	0.0608 (16)	−0.0203 (17)	0.0172 (16)	0.0074 (14)
O1	0.083 (2)	0.070 (2)	0.0420 (15)	−0.0189 (17)	0.0142 (15)	−0.0045 (14)
O2	0.090 (2)	0.0521 (18)	0.0466 (15)	−0.0224 (16)	0.0132 (15)	0.0016 (13)
O3	0.0694 (19)	0.0644 (18)	0.0293 (13)	−0.0207 (15)	0.0041 (13)	−0.0077 (12)
C1	0.086 (3)	0.074 (3)	0.049 (2)	−0.019 (3)	0.007 (2)	−0.022 (2)
C2	0.056 (3)	0.062 (3)	0.039 (2)	−0.015 (2)	0.0019 (19)	−0.005 (2)
C3	0.043 (2)	0.051 (2)	0.0352 (19)	−0.0133 (18)	−0.0034 (17)	−0.0077 (17)
C4	0.043 (2)	0.052 (2)	0.037 (2)	−0.0114 (18)	−0.0008 (17)	0.0014 (18)
C5	0.047 (2)	0.051 (2)	0.036 (2)	−0.0091 (18)	−0.0026 (18)	−0.0065 (18)
C6	0.041 (2)	0.054 (2)	0.0288 (18)	−0.0096 (18)	0.0004 (16)	−0.0030 (17)
C7	0.064 (3)	0.047 (2)	0.037 (2)	−0.018 (2)	−0.0015 (19)	0.0012 (18)
C8	0.053 (2)	0.046 (2)	0.043 (2)	−0.0099 (18)	−0.0010 (19)	−0.0058 (17)
C9	0.057 (3)	0.061 (3)	0.0338 (19)	−0.015 (2)	−0.0017 (18)	−0.0044 (19)
C10	0.041 (2)	0.061 (3)	0.0335 (19)	−0.0099 (19)	−0.0082 (17)	−0.0045 (18)
C11	0.066 (3)	0.069 (3)	0.041 (2)	−0.023 (2)	0.002 (2)	−0.013 (2)
C12	0.067 (3)	0.077 (3)	0.048 (2)	−0.015 (2)	−0.005 (2)	−0.028 (2)
C13	0.054 (3)	0.085 (3)	0.034 (2)	−0.011 (2)	0.0031 (19)	−0.011 (2)
C14	0.058 (3)	0.067 (3)	0.041 (2)	−0.010 (2)	−0.001 (2)	0.009 (2)
C15	0.054 (3)	0.065 (3)	0.038 (2)	−0.006 (2)	−0.0040 (19)	−0.0027 (19)
F3	0.135 (3)	0.0683 (19)	0.0704 (17)	−0.0089 (17)	0.0385 (17)	−0.0170 (14)
F4	0.118 (2)	0.0870 (19)	0.0553 (15)	0.0010 (16)	0.0135 (15)	0.0235 (14)
O4	0.091 (2)	0.078 (2)	0.0414 (16)	−0.0216 (18)	0.0155 (16)	−0.0122 (15)
O5	0.094 (2)	0.0533 (18)	0.0471 (16)	−0.0092 (16)	0.0153 (16)	−0.0126 (14)
O6	0.0694 (19)	0.0613 (18)	0.0321 (13)	−0.0113 (14)	0.0052 (13)	−0.0041 (13)
C16	0.090 (4)	0.081 (3)	0.044 (2)	−0.025 (3)	−0.002 (2)	0.010 (2)
C17	0.059 (3)	0.068 (3)	0.040 (2)	−0.023 (2)	−0.002 (2)	−0.005 (2)
C18	0.047 (2)	0.050 (2)	0.0326 (18)	−0.0149 (18)	−0.0043 (17)	−0.0038 (17)
C19	0.057 (3)	0.052 (2)	0.037 (2)	−0.018 (2)	−0.0007 (18)	−0.0101 (19)
C20	0.059 (3)	0.050 (2)	0.0332 (19)	−0.018 (2)	−0.0017 (18)	−0.0015 (17)
C21	0.048 (2)	0.052 (2)	0.0277 (18)	−0.0162 (19)	−0.0014 (16)	−0.0039 (17)
C22	0.057 (3)	0.053 (3)	0.041 (2)	−0.0044 (19)	−0.0054 (19)	−0.0074 (18)
C23	0.057 (3)	0.049 (2)	0.041 (2)	−0.0092 (19)	−0.0043 (19)	−0.0008 (18)
C24	0.057 (3)	0.057 (3)	0.035 (2)	−0.016 (2)	−0.0040 (18)	−0.0013 (18)
C25	0.040 (2)	0.063 (3)	0.0336 (19)	−0.0141 (19)	−0.0031 (16)	−0.0079 (18)
C26	0.064 (3)	0.055 (3)	0.038 (2)	−0.017 (2)	0.002 (2)	0.0002 (19)
C27	0.070 (3)	0.051 (3)	0.050 (2)	−0.011 (2)	0.010 (2)	−0.010 (2)
C28	0.060 (3)	0.076 (3)	0.032 (2)	−0.015 (2)	0.0041 (19)	−0.008 (2)
C29	0.065 (3)	0.063 (3)	0.038 (2)	−0.012 (2)	−0.005 (2)	0.012 (2)
C30	0.060 (3)	0.060 (3)	0.042 (2)	−0.006 (2)	−0.004 (2)	0.000 (2)

### Geometric parameters (Å, °)

**Table:**

F1—C12	1.349 (4)	F3—C27	1.358 (5)
F2—C14	1.357 (4)	F4—C29	1.358 (4)
O1—C2	1.234 (4)	O4—C17	1.245 (5)
O2—C4	1.353 (4)	O5—C19	1.352 (4)
O2—H2	0.8207	O5—H5	0.8205
O3—C6	1.356 (4)	O6—C21	1.354 (4)
O3—C9	1.421 (4)	O6—C24	1.426 (4)
C1—C2	1.501 (5)	C16—C17	1.491 (5)
C1—H1A	0.9600	C16—H16A	0.9600
C1—H1B	0.9600	C16—H16B	0.9600
C1—H1C	0.9600	C16—H16C	0.9600
C2—C3	1.456 (5)	C17—C18	1.456 (5)
C3—C8	1.391 (5)	C18—C23	1.396 (5)
C3—C4	1.416 (5)	C18—C19	1.410 (5)
C4—C5	1.380 (4)	C19—C20	1.379 (5)
C5—C6	1.362 (5)	C20—C21	1.383 (5)
C5—H5A	0.9300	C20—H20	0.9300
C6—C7	1.397 (5)	C21—C22	1.383 (5)
C7—C8	1.366 (4)	C22—C23	1.369 (5)
C7—H7	0.9300	C22—H22	0.9300
C8—H8	0.9300	C23—H23	0.9300
C9—C10	1.492 (5)	C24—C25	1.490 (4)
C9—H9A	0.9700	C24—H24A	0.9700
C9—H9B	0.9700	C24—H24B	0.9700
C10—C15	1.371 (5)	C25—C30	1.376 (5)
C10—C11	1.387 (5)	C25—C26	1.384 (5)
C11—C12	1.364 (5)	C26—C27	1.362 (5)
C11—H11	0.9300	C26—H26	0.9300
C12—C13	1.364 (5)	C27—C28	1.368 (5)
C13—C14	1.375 (5)	C28—C29	1.367 (6)
C13—H13	0.9300	C28—H28	0.9300
C14—C15	1.367 (5)	C29—C30	1.362 (5)
C15—H15	0.9300	C30—H30	0.9300
			
C4—O2—H2	109.6	C19—O5—H5	109.6
C6—O3—C9	117.5 (3)	C21—O6—C24	118.8 (3)
C2—C1—H1A	109.5	C17—C16—H16A	109.5
C2—C1—H1B	109.5	C17—C16—H16B	109.5
H1A—C1—H1B	109.5	H16A—C16—H16B	109.5
C2—C1—H1C	109.5	C17—C16—H16C	109.5
H1A—C1—H1C	109.5	H16A—C16—H16C	109.5
H1B—C1—H1C	109.5	H16B—C16—H16C	109.5
O1—C2—C3	120.9 (4)	O4—C17—C18	120.3 (4)
O1—C2—C1	119.1 (3)	O4—C17—C16	118.8 (3)
C3—C2—C1	120.0 (4)	C18—C17—C16	120.9 (4)
C8—C3—C4	116.5 (3)	C23—C18—C19	116.9 (3)
C8—C3—C2	123.5 (3)	C23—C18—C17	122.7 (4)
C4—C3—C2	119.9 (3)	C19—C18—C17	120.3 (4)
O2—C4—C5	117.6 (3)	O5—C19—C20	117.6 (4)
O2—C4—C3	121.1 (3)	O5—C19—C18	120.9 (3)
C5—C4—C3	121.3 (3)	C20—C19—C18	121.5 (4)
C6—C5—C4	120.2 (3)	C19—C20—C21	119.5 (4)
C6—C5—H5A	119.9	C19—C20—H20	120.2
C4—C5—H5A	119.9	C21—C20—H20	120.2
O3—C6—C5	124.9 (3)	O6—C21—C20	124.0 (3)
O3—C6—C7	115.0 (3)	O6—C21—C22	115.8 (3)
C5—C6—C7	120.1 (3)	C20—C21—C22	120.2 (3)
C8—C7—C6	119.6 (3)	C23—C22—C21	120.0 (4)
C8—C7—H7	120.2	C23—C22—H22	120.0
C6—C7—H7	120.2	C21—C22—H22	120.0
C7—C8—C3	122.4 (3)	C22—C23—C18	121.8 (4)
C7—C8—H8	118.8	C22—C23—H23	119.1
C3—C8—H8	118.8	C18—C23—H23	119.1
O3—C9—C10	109.5 (3)	O6—C24—C25	109.5 (3)
O3—C9—H9A	109.8	O6—C24—H24A	109.8
C10—C9—H9A	109.8	C25—C24—H24A	109.8
O3—C9—H9B	109.8	O6—C24—H24B	109.8
C10—C9—H9B	109.8	C25—C24—H24B	109.8
H9A—C9—H9B	108.2	H24A—C24—H24B	108.2
C15—C10—C11	119.1 (3)	C30—C25—C26	118.7 (3)
C15—C10—C9	122.8 (3)	C30—C25—C24	118.8 (4)
C11—C10—C9	118.1 (3)	C26—C25—C24	122.5 (3)
C12—C11—C10	119.2 (4)	C27—C26—C25	119.3 (4)
C12—C11—H11	120.4	C27—C26—H26	120.4
C10—C11—H11	120.4	C25—C26—H26	120.4
F1—C12—C11	119.1 (4)	F3—C27—C26	118.6 (4)
F1—C12—C13	117.5 (4)	F3—C27—C28	117.7 (3)
C11—C12—C13	123.4 (4)	C26—C27—C28	123.7 (4)
C12—C13—C14	115.6 (3)	C29—C28—C27	115.1 (3)
C12—C13—H13	122.2	C29—C28—H28	122.4
C14—C13—H13	122.2	C27—C28—H28	122.4
F2—C14—C15	118.8 (4)	F4—C29—C30	118.7 (4)
F2—C14—C13	117.7 (3)	F4—C29—C28	117.4 (3)
C15—C14—C13	123.5 (4)	C30—C29—C28	124.0 (4)
C14—C15—C10	119.1 (4)	C29—C30—C25	119.2 (4)
C14—C15—H15	120.5	C29—C30—H30	120.4
C10—C15—H15	120.5	C25—C30—H30	120.4
			
O1—C2—C3—C8	179.7 (4)	O4—C17—C18—C23	178.5 (4)
C1—C2—C3—C8	1.2 (6)	C16—C17—C18—C23	−0.8 (6)
O1—C2—C3—C4	−2.9 (6)	O4—C17—C18—C19	−2.0 (6)
C1—C2—C3—C4	178.6 (4)	C16—C17—C18—C19	178.6 (4)
C8—C3—C4—O2	180.0 (3)	C23—C18—C19—O5	−179.4 (3)
C2—C3—C4—O2	2.4 (5)	C17—C18—C19—O5	1.2 (6)
C8—C3—C4—C5	−0.7 (5)	C23—C18—C19—C20	1.5 (5)
C2—C3—C4—C5	−178.2 (3)	C17—C18—C19—C20	−178.0 (3)
O2—C4—C5—C6	−179.9 (3)	O5—C19—C20—C21	−179.9 (3)
C3—C4—C5—C6	0.7 (6)	C18—C19—C20—C21	−0.7 (6)
C9—O3—C6—C5	0.7 (5)	C24—O6—C21—C20	0.5 (5)
C9—O3—C6—C7	−179.6 (3)	C24—O6—C21—C22	179.9 (3)
C4—C5—C6—O3	178.9 (3)	C19—C20—C21—O6	178.9 (3)
C4—C5—C6—C7	−0.8 (5)	C19—C20—C21—C22	−0.6 (6)
O3—C6—C7—C8	−178.9 (3)	O6—C21—C22—C23	−178.5 (3)
C5—C6—C7—C8	0.8 (6)	C20—C21—C22—C23	1.0 (6)
C6—C7—C8—C3	−0.8 (6)	C21—C22—C23—C18	−0.1 (6)
C4—C3—C8—C7	0.7 (5)	C19—C18—C23—C22	−1.1 (5)
C2—C3—C8—C7	178.2 (4)	C17—C18—C23—C22	178.4 (4)
C6—O3—C9—C10	178.5 (3)	C21—O6—C24—C25	178.4 (3)
O3—C9—C10—C15	2.3 (5)	O6—C24—C25—C30	−179.6 (3)
O3—C9—C10—C11	−178.3 (3)	O6—C24—C25—C26	0.1 (5)
C15—C10—C11—C12	−0.5 (6)	C30—C25—C26—C27	−0.9 (6)
C9—C10—C11—C12	−180.0 (4)	C24—C25—C26—C27	179.5 (4)
C10—C11—C12—F1	179.8 (4)	C25—C26—C27—F3	−179.6 (4)
C10—C11—C12—C13	1.2 (7)	C25—C26—C27—C28	0.5 (7)
F1—C12—C13—C14	179.8 (4)	F3—C27—C28—C29	−180.0 (4)
C11—C12—C13—C14	−1.6 (6)	C26—C27—C28—C29	−0.1 (7)
C12—C13—C14—F2	179.9 (4)	C27—C28—C29—F4	179.9 (4)
C12—C13—C14—C15	1.5 (6)	C27—C28—C29—C30	0.0 (6)
F2—C14—C15—C10	−179.4 (4)	F4—C29—C30—C25	179.7 (4)
C13—C14—C15—C10	−1.0 (6)	C28—C29—C30—C25	−0.3 (7)
C11—C10—C15—C14	0.5 (6)	C26—C25—C30—C29	0.8 (6)
C9—C10—C15—C14	179.9 (4)	C24—C25—C30—C29	−179.6 (4)

### Hydrogen-bond geometry (Å, °)

**Table:**

*D*—H···*A*	*D*—H	H···*A*	*D*···*A*	*D*—H···*A*
O2—H2···O1	0.82	1.81	2.533 (4)	147.
O5—H5···O4	0.82	1.80	2.525 (4)	147.
C8—H8···O5	0.93	2.49	3.382 (5)	161.
C13—H13···O1^i^	0.93	2.44	3.342 (5)	165.
C28—H28···O4^ii^	0.93	2.40	3.315 (5)	168.

Symmetry codes: (i) *x*+1, *y*, *z*−1; (ii) *x*−1, *y*, *z*+1.

## References

[bb1] Dermer, O. C. (1934). *Chem. Rev.* **14**, 385–430.

[bb2] Ma, Y.-T., Wang, J.-J., Liu, X.-W., Yang, S.-X. & Gao, J.-M. (2010). *Acta Cryst.* E**66**, o52. 10.1107/S1600536809051411 PMC2980010 21580155

[bb3] Sheldrick, G. M. (1996). *SADABS* University of Göttingen, Germany.

[bb4] Sheldrick, G. M. (2008). *Acta Cryst.* A**64**, 112–122. 10.1107/S0108767307043930 18156677

[bb5] Siemens (1996). *SMART* and *SAINT* Siemens Analytical X-ray Instruments Inc., Madison, Wisconsin, USA.

